# Improving adherence to colorectal cancer surveillance guidelines: results of a randomised controlled trial

**DOI:** 10.1186/s12885-017-3095-x

**Published:** 2017-02-06

**Authors:** Mariko Carey, Robert Sanson-Fisher, Finlay Macrae, Emilie Cameron, David Hill, Catherine D’Este, Christopher Doran

**Affiliations:** 1 Priority Research Centre for Health Behaviour (PRCHB), School of Medicine & Public Health, University of Newcastle, W4, HMRI Building, Callaghan, NSW Australia; 2 Hunter Medical Research Institute (HMRI), New Lambton, NSW Australia; 3 Cancer Council Victoria, Melbourne, VIC Australia; 4 Department of Colorectal Medicine and Genetics, The Royal Melbourne Hospital, Melbourne, VIC Australia; 5 Melbourne School of Population & Global Health Melbourne School of Psychological Sciences, The University of Melbourne, Parkville, Melbourne, VIC 3010 Australia; 6 National Centre for Epidemiology and Population Health, Research School of Population Health, Australian National University, Canberra, ACT Australia; 7 School Human, Health and Social Sciences, Central Queensland University, Brisbane, QLD Australia

**Keywords:** Colorectal cancer, Surveillance, Colonoscopy, Bowel cancer, Guideline adherence, Implementation science, Randomized controlled trial

## Abstract

**Background:**

Colorectal cancer (CRC) survivors are at increased risk of developing the disease again. Surveillance guidelines are aimed at maximising the early detection of recurring or new cancers and pre-cancerous polyps. The frequency and type of surveillance recommended depends on the type of treatment for the initial CRC, the extent of colonoscopic investigation prior to treatment and the results of previous surveillance tests. This paper aimed to test the effect of a paper–based educational intervention to improve adherence to colonoscopy following treatment for colorectal cancer.

**Methods:**

People with a diagnosis of colorectal cancer within the last 10 months, aged ≥18 and English speaking were recruited through a population-based cancer registry in Australia. Participants were randomly allocated to either the intervention or control. Participants completed an interview at baseline. Self-reported participation in colonoscopy was obtained at 12 month followup by survey. Those allocated to the control received a generic pamphlet on colorectal cancer treatment; while intervention participants received a letter which provided specific information about guideline recommendations for surveillance colonoscopy. Rates of guideline adherence were compared between groups. The guideline recommendations for the timing of surveillance colonoscopy changed part way through the study. This change occurred after all intervention materials had been sent, but prior to all participants completing the 12 month follow up. Post hoc analyses were conducted to assess adherence to the new guidelines.

**Results:**

Of the 767 participants, 604 (79%) had had surgery, had stage I – III disease and completed the baseline interview within 12 months of diagnosis (intervention = 305; control = 299). There was no significant difference between those adherent to surveillance colonoscopy guidelines, in the control (67, 27%) and intervention groups (80, 31%) at followup (difference = 4.3% (95%CI:-3.7%, 12%), *χ* ^2^(1df) = 1.09, *P* = 0.296). Overall, 246 (49%) participants were adherent to the new guidelines, compared to 147 (29%) adherent to the old guidelines.

**Conclusions:**

Results indicate the paper-based educational intervention is not effective in improving adherence to colorectal cancer surveillance guidelines for colonoscopy.

**Trial registration number:**

ACTRN12609000628246 Registration date: 28/07/2009

**Electronic supplementary material:**

The online version of this article (doi:10.1186/s12885-017-3095-x) contains supplementary material, which is available to authorized users.

## Background

### Surveillance guideline recommendations

Colorectal cancer (CRC) survivors are at increased risk of developing the disease again [1–3]. Surveillance guidelines are aimed at maximising the early detection of recurring or new cancers and pre-cancerous polyps [3]. The frequency and type of surveillance recommended depends on the type of treatment for the initial CRC, the extent of colonoscopic investigation prior to treatment and the results of previous surveillance tests. In Australia, National Health and Medical Research Council (NHMRC) guidelines released in 1999 [1] and 2005 [3] recommended that those who did not have a colonoscopy when first diagnosed should have one 3–6 months after surgery then every 3–5 years. In December 2011 the guidelines specific to surveillance colonoscopy [4] were updated to include a colonoscopy one year after surgery unless a complete post-operative colonoscopy has been performed previously.

### Adherence to surveillance guideline recommendations

One Australian study indicated only 23% of surveillance colonoscopies were in accordance with NHMRC recommendations, with the screening interval shorter than recommended in 70% of non-adherent cases [1, 5]. Similarly, a study in the USA [6] found that around 30% of CRC survivors had subsequent colonoscopies within two years of a normal result, more frequently than is recommended by guidelines. In contrast, another US study [7] found that only 49% of CRC survivors received a colonoscopy within 14 months of surgery. Endoscopists’ recommendation for early follow up has been associated with overuse of surveillance colonoscopy [8]. However studies indicate that specialists often do not know the recommended surveillance interval [9, 10] and those who are aware of the recommendations often do not follow them [11].

### Efforts to increase adherence to guidelines

Despite evidence of variable adherence to surveillance colonoscopy guidelines [1, 6, 7], there has been little research on interventions to promote adherence to guidelines for CRC follow up. There have, however, been some evaluations of strategies designed to improve adherence to surveillance colonoscopy in non-cancer samples (e.g. post polypectomy). For example, an improvement in guideline adherence was demonstrated at a single site following dissemination of guidelines to all specialists and implementation of a nurse co-ordinator role to ensure that surveillance recommendations matched guidelines [5]. Physician reminders were shown to increase surveillance colonoscopy adherence following adenoma removal in a randomised controlled trial (RCT) involving 358 patients [12].

Given the limited research on interventions to promote adherence to surveillance colonoscopy recommendations, we aimed to investigate the impact of a centrally delivered paper-based intervention to improve adherence to surveillance colonoscopy recommendations following curative resection for CRC. The intervention approach selected was designed to be low cost, and have potential for broad reach to the entire affected population, rather than being focussed on a particular health care service.

### Aims

The aim of the study was 1) to determine the effectiveness of a print-based intervention to increase the proportion of CRC patients who undertake surveillance tests in line with NHMRC recommendations in the year following the intervention; and 2) to examine socio-demographic characteristics and disease characteristics associated with appropriate screening of CRC patients.

## Methods

### Setting and approvals

Participants were identified through a population-based cancer registry in Victoria, Australia. Ethical approvals were obtained from the University of Newcastle (H-2008-0047) and the Cancer Council Victoria’s Human Research Ethics Committees (HREC-0810) and written informed consent was obtained from all participants. Data were collected between February 2010 and November 2012. This study was part of a larger trial in which people with CRC were able to invite their first degree relatives to participate in a study of adherence to CRC screening among first degree relatives [13]. Only results related to index cases are reported here.

### Participants

People with CRC were invited to participate if they met the following criteria: 1) aged 18 or older; 2) within 10 months of a primary invasive CRC diagnosis registered with the cancer registry (ICD-10: C18, C19 and C20); 3) considered able to participate by their clinician and 4) sufficiently fluent in English to complete and understand the consent form and study surveys. Only those participants who completed a baseline interview within 12 months of diagnosis, had surgery for CRC and did not have Stage IV cancer were included in this study.

### Procedure

**Fig. 1:**
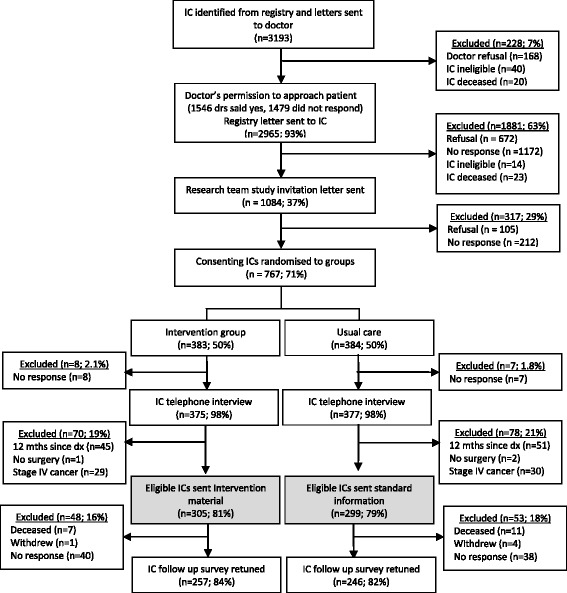
Flowchart of recruitment of index cases (IC) to the study

The recruitment process is shown in Fig. 1. A letter was sent to the notifying clinician of each potentially eligible person identified by the registry. Clinicians were asked to advise the registry within a month if the patient was unsuitable to be approached. Eligible patients were then invited by the registry to provide written consent for their contact details released to the research team. Those who agreed were mailed an invitation to participate in the trial. Up to two reminder letters were sent after four weeks to non-responders.

Participants were randomised to either the intervention or control group using a centrally-managed computer-generated randomisation procedure.

Participants completed a baseline computer-assisted telephone interview (CATI; Additional file 1) and were asked to provide contact details for their general practitioner and/or specialist (surgeon, oncologist and/or gastroenterologists). A paper and pencil follow up survey (Additional file 2) was sent to participants 12 months after the baseline interview. Up to two reminder surveys were sent to those who had not responded within 4 weeks.

### Experimental groups

#### Control

Following the baseline interview, participants randomised to the control group were mailed a generic pamphlet on CRC.

#### Intervention

Participants randomised to the intervention group received a letter detailing recommendations for follow-up care. The letter highlighted NHMRC [3] recommendations: 1) to visit a doctor every 3–6 months for 2 years after surgery; 2) that most patients will need to have a colonoscopy every 3–5 years. However those who did not have a complete colonoscopy when first diagnosed should have one 3–6 months after surgery; 3) a sigmoidoscopy may be arranged if the cancer was in the rectum; and 4) other tests may be scheduled including blood tests and Computed Tomography (CT) scans. The patient’s nominated general practitioner and cancer specialist were also sent a fact sheet with information about best evidence surveillance care.

### Measures

The following data were collected at baseline: 1) *Registry data:* Diagnosis date, age at diagnosis, sex, postcode, cancer type and cancer staging were provided by the registry for each consenting participants. Grouped de-identified data on these characteristics were provided for non-consenters to allow assessment of consent bias; 2) *Sociodemographic characteristics:* Participants self- reported their age, marital status, highest level of education, employment, health insurance status; 3) *Cancer treatments:* Participants were asked whether they had surgery for bowel cancer and when their surgery was performed; 4) *Participation in bowel cancer tests:* participants were asked whether they had ever had one of the following tests for bowel cancer: colonoscopy, Faecal Occult Blood Test (FOBT), or sigmoidoscopy. Brief descriptions of each test were provided to aid accurate recall. Those who responded affirmatively were asked additional questions about the timing of their most recent test. Response options included: 1, 2, 3, 4, 5, 6, 7, 8, 9, 10, 11, 12 months ago, 1–2 years ago, 2–5 years ago, over 5 years ago.

At 12 months follow up, participants were asked to provide self-reported data on how many times in the past year they had seen their cancer specialist and general practitioner for bowel cancer follow up care, whether they had a colonoscopy/FOBT/sigmoidoscopy and if so in which month they had the test.

### Primary outcome: adherence to surveillance guidelines for colonoscopy

Self-report data on colonoscopy was used to assess adherence. We allowed 1 month leeway to have the procedure. Participants were considered adherent to surveillance guidelines if they reported having a colonoscopy at any time from four months prior to their diagnosis date up to 8 months after their surgery date; otherwise they were classified as not adherent. Those classified as non-adherent were considered underscreened if they reported no colonoscopies in this period or overscreened if they reported a subsequent colonoscopy up to 2 years after diagnosis.

### Statistical methods

Participant demographic and disease characteristics are presented for the control and intervention groups. The primary outcome, adherence to surveillance guidelines at 12 months follow up, was compared between the control and intervention groups using a chi-squared test. The primary analysis was performed as a complete case analysis, with sensitivity analyses using multiple imputation for missing data. Imputation was undertaken with 20 datasets using a separate monotone imputation model for the intervention and control groups which included the variables age category, sex, rurality, education, employment, whether Australian born, relationship status, insurance status, cancer stage (TNM), cancer site and timing of most recent colonoscopy recorded in the baseline interview to impute adherence at follow up and follow up specialist visits. Estimates were then obtained using Rubin’s rules [14]. A logistic regression model was used to examine the characteristics associated with adherence to surveillance guidelines compared to those who were not adherent, while adjusting for potential confounders. The factors included in the model were age, sex, location, Australian born, education, employment, private health insurance, cancer location, cancer stage and follow-up specialist visits. This analysis was also undertaken on the multiple imputed data. Analyses were conducted in Stata 11.2 [15].

Assuming 50% adherence in the control group, a loss to followup of 20%, and a 5% significance level, a sample of 1200 patients (600 per group) would have at least 80% power to detect a difference in adherence of 10% between groups at 12 month follow up (this was considered to be a clinically important effect).

### Effect of guideline change

New guidelines for surveillance colonoscopy were introduced in December 2011 [4] recommending that colonoscopy should be performed one year after curative resection for CRC, unless a complete post-operative colonoscopy has been performed sooner. When these guidelines were released, all intervention materials had been sent out to the intervention participants, however, some participants had not yet completed the 12 month follow up survey. To enable a post hoc investigation of the adoption of the new guidelines we compared the proportion of participants who reported having a colonoscopy in the follow up period among those who completed the follow up survey before 31^st^ December 2011 and those who returned the survey after 30 March 2012, using the Chi-squared test (Those returning a follow up survey in January to February 2012 were excluded from the analysis in order to provide a bedding down period between the two sets of guidelines). We calculated adherence to the new guidelines (colonoscopy in the year following surgery) for the entire sample. We have provided examples of how adherence status would be classified using the old and new guidelines in Table 1.

**Table 1:** Comparison of adherence classification using the old and new guidelines

	Guidelines recommendations	
Examples	Old guidelines: Colonoscopy that visualises the entire colon should be performed at the time of diagnosis^a^. If this is not possible, colonoscopy should be performed within 3–6 months of surgery. Colonoscopy should be performed 3–5 years after surgery.	New guidelines: Colonoscopy should be performed one year after the resection, unless a complete post-operative colonoscopy has been performed sooner.
	Classification of adherence	
Colonoscopy at diagnosis; no colonoscopy reported at 12 month follow up	Adherent	Non-adherent
Colonoscopy at diagnosis; followed by colonoscopy 12 months later	Non-adherent	Adherent
Colonoscopy at diagnosis, and then another colonoscopy within 6 months of surgery	Adherent	Adherent
Colonoscopy 6 months post surgery; no subsequent colonoscopy reported at 12 month follow up	Adherent	Adherent
Colonoscopy 6 months post surgery; subsequent colonoscopy reported at 12 month follow up	Non-adherent	Non-adherent

^a^We were unable to tell if the colonoscopy performed at diagnosis visualised the entire colon. Therefore, any cases where this criteria was crucial to determining adherence were classified as adherent. For example if a colonoscopy was performed at baseline and then again at 6 months, we assumed that the diagnostic colonoscopy did not enable visualisation of the entire colon, and therefore classified the patient as adherent

## Results

### Sample characteristics

From 3193 people with CRC identified by the registry, 168 were considered ineligible by their notifying clinicians, 672 did not provide consent to be contacted by the research team, 43 were deceased, 54 were ineligible and 1172 did not respond (see Fig. 1). Of the remaining 1084 who were approached to participate in the study, 752 (69%; 25% of eligible individuals) consented to participate and completed the baseline interview. After removing those who did not have surgery, had Stage IV cancer or did not complete the baseline interview within a year of being diagnosed, the sample consisted of 604 participants; Of which 305 were randomised to the intervention group and 299 to the control group. The characteristics of the sample are shown in Table 2 and appear similar for the two groups. 503 (83%) participants returned a follow up survey sent out 1 year after the baseline survey (257, 84% in the intervention group and 246, 82% in the control group). The follow up surveys were returned up to 3 years from diagnosis.

**Table 2:** Characteristics of the sample

Variable	Control	Intervention
	*N* = 299	*N* = 305
	*n* (%)	*n* (%)
Age (*n* = 653)	Mean = 66 (SD = 11)	Mean = 68 (SD = 12)
under 50	16 (5%)	18 (6%)
50–59	60 (20%)	55 (18%)
60–69	98 (33%)	83 (27%)
70–79	85 (28%)	81 (27%)
over 80	40 (13%)	68 (22%)
Male (*n* = 653)	171 (57%)	154 (50%)
Married/defacto (*n* = 653)	230 (77%)	205 (67%)
Born in Australia (*n* = 591)	205 (77%)	223 (80%)
Urban dwelling (*n* = 652)	202 (68%)	194 (64%)
Education (*n* = 651)		
University or vocational training	99 (33%)	200 (33%)
Secondary school completed	44 (14%)	87 (14%)
Secondary school not completed	161 (53%)	315 (52%)
Employed (*n* = 653)	106 (32%)	98 (34%)
Private health insurance (*n* = 653)	197 (66%)	201 (66%)
Previous cancer diagnosis (*n* = 652)	33 (11%)	54 (18%)
Left-sided tumour (*n* = 649)	177 (59%)	162 (54%)
Disease stage (*n* = 652)		
TNM Stage I	95 (32%)	98 (33%)
TNM Stage II	100 (34%)	115 (38%)
TNM Stage III	99 (34%)	88 (29%)
Returned a follow up survey (*n* = 653)	246 (82%)	257 (84%)
Died in follow-up period	11 (4%)	7 (2%)
Days between diagnosis and Intervention (*n* = 653)	Median = 219 (IQR:171-281)	Median = 226 (IQR:189-281)

### Primary outcome: adherence to surveillance colonoscopy guidelines

There was no significant difference between those adherent to surveillance colonoscopy guidelines, in the control (67, 27%) and intervention groups (80, 31%) at followup (difference = 4.3% (95%CI: -3.7%, 12%), *χ* ^2^(1df) = 1.09, *P* = 0.296; Table 3). This result did not change under a multiple Imputation model for missing outcome data (Odds ratio = 1.25 (0.86, 1.81); *P* = 0.234). Table 3 shows the proportion of participants adherent to guidelines at followup, under screened and over screened in the control and intervention groups. Overall in the 1–3 years following surgery for CRC, 147 (29%) participants reported receiving surveillance colonoscopies in line with guidelines (118 (24%) perioperatively, 29 (5.8%) up to 8 months since surgery); 27 (5.4%) were underscreened (16 (3.2%) reported a colonoscopy outside of the recommended time frame and 11 (2.2%) reported not having any colonoscopies) and 326 (65%) had more colonoscopies than was recommended (over screened). The majority of overscreening was due to having a colonoscopy at diagnosis, followed by another within 12 to 14 months (*n* = 197; 39%). This pattern of overscreening is consistent with the new guideline recommendations.

**Table 3:** Adherence to surveillance guidelines in the intervention and control groups

	Intervention (*N* = 270) *n* (%)	Control (*N* = 259) *n* (%)	Total (*N* = 529) *n* (%)
Adherent at follow up	80(32%)	67(27%)	147(29%)
Non-adherent at follow up	174(69%)	179(73%)	353(71%)
Under screened	10(3.9%)	17(6.9%)	27(5.4%)
Over screened	164(65%)	162(66%)	326(65%)

### Characteristics associated with adherence

Table 4 shows the results of multiple logistic regression of factors associated with adherence at follow up compared to non-adherence. Participants had higher odds of being adherent to surveillance recommendations if they had a left-sided tumour relative to a transverse or right sided tumour; did not see a specialist for follow up care relative to those who did; or were over 80 compared with younger age groups. Sensitivity analysis using multiple imputation provided similar results to complete case analysis.

**Table 4:** Multiple logistic regression of factors associated with adherence to surveillance guidelines at follow up compared to those who were over screened (*n* = 455)

Variable	Adherent to surveillance guidelines	Odds ratio	*P* value*
		(95% CI)	
Experimental group			
Control	55 (26%)	REF	
Intervention	66 (29%)	1.01 (0.64–1.6)	0.954
Age			
under 50	3 (14%)	0.44 (0.11–1.83)	
50–59	14 (17%)	0.56 (0.24–1.34)	
60–69	31 (22%)	0.77 (0.42–1.4)	
70–79	39 (31%)	REF	
over 80	34 (47%)	2 (1.05–3.98)	0.049
Sex			
Male	68 (29%)	REF	
Female	53 (26%)	0.91 (0.56–1.47)	0.697
Highest level of education			
University or vocational training	34 (22%)	REF	
Secondary school completed	23 (34%)	1.6 (0.8–3.12)	
Secondary school not completed	64 (29%)	1.03 (0.6–1.77)	0.367
Relationship status			
Single	36 (33%)	REF	
Married/defacto	85 (26%)	1.01 (0.58–1.76)	0.97
Employment status			
Not employed	95 (33%)	REF	
Employed	26 (17%)	0.58 (0.3–1.12)	0.107
Australian born			
No	24 (26%)	REF	
Yes	97 (28%)	1.2 (0.68–2.14)	0.519
Location or residence			
Rural	52 (34%)	REF	
Urban	69 (24%)	0.65 (0.4–1.05)	0.081
Private health insurance			
No	44 (34%)	REF	
Yes	77 (25%)	0.81 (0.49–1.33)	0.406
Disease stage			
TNM Stage I	45 (31%)	REF	
TNM Stage II	44 (27%)	0.67 (0.39–1.16)	
TNM Stage III	32 (24%)	0.76 (0.43–1.36)	0.349
Cancer site			
Right sided	45 (24%)	REF	
left-sided	76 (30%)	1.8 (1.13–3.02)	0.014
Saw specialist for follow up care			
No	14 (61%)	REF	
Yes	107 (26%)	0.35 (0.14–0.91)	0.031

* *P* value for adjusted Wald test

### Effect of guideline change

Overall, 246 (49%) participants were adherent to the new guidelines, compared to only 147(29%) who were adherent to the old guidelines. According to the new guidelines, 24 (4.8%) were overscreened and 230 (46%) were underscreened. Those who returned a follow up survey after the guideline change (*n* = 164) were significantly more likely to have had a colonoscopy in the follow up period (124, 76%) than those who returned a follow up survey prior (*n* = 180) to the guideline change (117, 65%) (*χ* ^2^(1df) = 4.61; *P* = 0.032) excluding those who returned a follow up survey between Dec 2011 and March 2012 (*n* = 159).

## Discussion

Our intervention had no impact on adherence to CRC surveillance colonoscopies. In contrast, previous studies have indicated that more intensive approaches targeting clinicians via point of care reminders or feedback on guideline adherence are effective at improving adherence [5, 12]. Behavioural change theories indicate that there are a range of factors that can influence adoption of evidence in clinical practice. These include knowledge, skills and beliefs, professional role and identity, environmental context and resources, memory and attentional, social factors, emotion and goal setting [16]. Complex behaviours, for example, lifestyle changes such as quitting smoking or changing one’s diet are likely to require complex interventions which target a range of these factors. In the case of one-off or intermittent behaviours such as participation in cancer screening simpler interventions such as mail-based invitations have been shown to be effective [17]. Therefore, on face value it seems that a simple mail-based intervention could be effective for improving adherence to colorectal cancer surveillance colonoscopy recommendations. However, there are several factors that may explain why this type of intervention is often effective at promoting cancer screening uptake but was not effective at improving surveillance adherence. First, our study did not seek to improve uptake of surveillance colonoscopy but rather to improve adherence. Screening uptake may be easier to achieve because this is largely consistent with the beliefs of patients regarding early detection being beneficial. In contrast, reducing the use of surveillance tests may create anxiety about a potential recurrence being missed [18]. Similarly, clinicians may have concerns about missing a recurrence and /or may be motivated to reassure patients through the ordering of such tests.

Therefore, it is possible that our paper- based intervention was not intensive enough and did not adequately address these barriers. Of further concern, the low participation rates (25%) indicate that the approach used, regardless of effect, is unlikely to be a feasible method to reach the target group.

### Early guideline adoption?

Our post hoc analyses showed that a significantly greater proportion of respondents were adherent to the new guidelines (49%) compared to the guidelines that were current during the majority of the study period (29%). This is despite the fact that the latter guideline recommendations had been in place for over 10 years at the time our study commenced [1, 3]. At the time our study was being implemented, it is possible that some clinicians were aware of the planned changes to the guidelines and had already adapted their practice to suit these. A prior Australian study examining guideline adherence to surveillance colonoscopy between 1989 and 2001 also demonstrated poor adherence to guideline recommendations. Most notably the rate of adherence increased from 20% to just 23% following the initial release of the guidelines in 1999. Similar to our study, overscreening (i.e. receiving surveillance colonoscopy too early) was the most common reason for nonadherence [1]. Taken together, these data suggest that while these guidelines had been in place for over 10 years, adherence rates remained low. Given this, it appears that the most likely explanation for the higher adherence to the new guidelines is that they were more closely aligned with practice patterns over the past 20 years.

The proportion of respondents reporting a colonoscopy in the follow up period of our study increased following the release of the updated guideline in late 2011. While it is difficult to compare the adherence rates reported in the current study with those of Yusoff [1] due to method differences, the 20% increase in adherence reported in our study can be contrasted with only a 3% increase over a similar time period following the release of the previous guidelines. Likewise, based on the old guideline recommendations, comparison of the adherence rates reported in Yusoff’s data of 23% in 1999–2001 and our data from 2010 to 2012 indicates an increase in adherence of 6% from 23% to just 29 over this 13 year time period. Again this rate of change is compared to the 20% change over the 3 year period of our study, the magnitude and rapidity of practice change attributable to the change in guideline recommendations can be appreciated. Prior research has shown that adoption is more likely to occur when recommendations are aligned with existing beliefs [19]. Indeed, this rapid change of practice suggests that the new recommendations are more consistent with clinicians’ beliefs regarding best practice.

### Limitations

While the participation rates achieved in our study were similar to those in other studies conducted through cancer registries [20, 21], they limit the extent to which our findings are generalizable to the broader population of people with CRC. Further, the overall sample size obtained was lower than anticipated resulting in reduced statistical power, which was not completely compensated for by lower than expected adherence in the control group. Post hoc power analysis suggests that we would have had 80% power to detect approximately 12.5% difference in adherence between groups.

We relied on patient self-reported colonoscopy use and timing, so it is possible that the data were subject to biases in recall and/ or social desirability. Self-reporting on *when* a colonoscopy was done may have been particularly problematic for some participants in the study. Unfortunately, accessing medical records data or Medicare (claims) data was not considered feasible for a population study such as ours given the number of different health care providers involved in the care of the patients, and inconsistent availability of Medicare data depending upon treatment setting. We did not collect detailed information about current treatment or disease progression therefore it is possible that for some people in the sample the surveillance guidelines may not have been appropriate. We excluded those with Stage IV cancer to limit the effect of this. At the commencement of our study, we did not anticipate the guideline change, and so did not collect additional data on clinician beliefs to aid in our interpretation of the factors influencing the practice patterns observed.

## Conclusions

Our results indicate that a paper-based educational intervention aimed at patients and surgeons is not effective in improving adherence to CRC surveillance guidelines for colonoscopy. Notwithstanding recent changes to the guideline recommendations regarding the timing of colonoscopy, poor adherence to surveillance colonoscopy recommendations was observed. However, higher rate of adherence to the new guideline recommendations released during our study, suggests that the new guidelines are more consistent with existing practice patterns.

## Additional files

Additional file 1: Interview Guide for computer-assisted telephone interview (CATI) completed by participants at baseline. Description: Questions from the baseline CATI guide used in the research presented. (PDF 31 kb)
Additional file 2: Follow up survey sent to participants 12 months after the baseline interview. Description: Questions from the pen and paper follow up survey used in the research presented. (PDF 24 kb)

## Abbreviations

CATI: Computer-assisted telephone interview
CRC: Colorectal cancer
CT: Computed Topography
FOBT: Faecal Occult Blood Test
NHMRC: National Health and Medical Research Council
RCT: Randomised controlled trial
TNM: Tumour Node Metastasis system of describing cancer stage
