# Supplementary material for: Improving adherence to colorectal cancer surveillance guidelines: results of a randomised controlled trial
Source: BMC Cancer. 2017 Feb 6;17:106. doi: 10.1186/s12885-017-3095-x (PMC5294678; doi:10.1186/s12885-017-3095-x)
Supplement: Additional file 1: — Interview Guide for computer-assisted telephone interview (CATI) completed by participants at baseline. Description: Questions from the baseline CATI guide used in the research presented. (PDF 31 kb) [file 12885_2017_3095_MOESM1_ESM.pdf]

Baseline CATI Guide

Personal history of bowel cancer

I will now ask about your experiences of treatment and follow up care for bowel cancer.

1) Prior to this current diagnosis, had you ever been diagnosed with bowel cancer?

- Yes
- No

2) How did you come to be diagnosed with bowel cancer?

Record response that is closest

- Routine screening (I had no symptoms)
- Routine diagnostic test (which I completed because I had symptoms)
- Other – please describe

____

____

3) Was there more than one bowel cancer tumour found when you were diagnosed?

- Yes
- No
- Don't know/Not sure

4) Did you have surgery for bowel cancer? *If yes*, **what was the purpose of the surgery? (***Select all that apply)*

- Did not have surgery go to 7
- To see if I definitely had cancer go to 6
- To remove the cancer/cure the cancer go to 5
- To see what stage the cancer was go to 6
- To improve the symptoms of cancer go to 6

5) As far as your surgeon could tell, was all the cancer removed during surgery?

☐ Yes

☐ No

☐ Don’t know/Not sure

6) How long ago did you have surgery? *If the person has had more than one surgery prompt for surgery to remove bowel cancer. If the person did not have surgery to remove the cancer, then prompt for most recent surgery.*

☐ 1 month

☐ 2 months

☐ 3 months

☐ 4 months

☐ 5 months

☐ 6 months

☐ 7 months

☐ 8 months

☐ 9 months

☐ 10 months

☐ 11 months

☐ 12 months

In the information sheet we sent there is a list of tests for which can be used in bowel cancer screening and follow-up care. Have you got that in front of you now? *(If no, arrange to call back or send an additional copy).*

7) Have you ever had any of those tests?

☐ Yes	go to 8
☐ No	go to 11
☐ Don’t know/Not sure	go to 11

8) Which tests have you had? *Select all that apply*

- ☐ Colonoscopy
- ☐ Sigmoidscopy
- ☐ Faecal Occult Blood Test (FOBT) or Faecal Immunochemical Test (iFIT)

Repeat for ***<u>each</u>*** *test indicated as yes:*

9) How many (insert test name) have you had?

- ☐ 1
- ☐ 2
- ☐ 3
- ☐ 4 or more

How long ago was the most recent test undertaken? (*if the person has had more than one test, ask them to answer in relation to their most recent test*).

- ☐ 1 month
- ☐ 2 months
- ☐ 3 months
- ☐ 4 months
- ☐ 5 months
- ☐ 6 months
- ☐ 7 months
- ☐ 8 months
- ☐ 9 months
- ☐ 10 months
- ☐ 11 months
- ☐ 12 months
- ☐ More than 12 months but less than 2 years ago
- ☐ More than 2 years but less than 5 years ago
- ☐ More than 5 years ago

*Repeat for **each** test indicated as yes:*

10) Why did you have the test?

- Don’t know/not sure
- To see if surgery had removed all the bowel cancer
- To see if the cancer had spread
- To see if the cancer had come back
- To test whether I definitely had bowel cancer/diagnose bowel cancer
- To screen for bowel cancer

For people who indicated that they have had more than one test

11. Why did you have the test before the most recent one?

- Don’t know/not sure
- To see if surgery had removed all the bowel cancer
- To see if the cancer had spread
- To see if the cancer had come back
- To test whether I definitely had bowel cancer/diagnose bowel cancer
- To screen for bowel cancer

12) Looking at the list of tests again. Are you booked in to have any of these tests as part of your follow up care?

Yes	go to 12
No	go to Module F
Don’t know/Not sure	go to Module F

13) Which tests are you booked in for?

- Colonoscopy
- Sigmoidoscopy
- Faecal Occult Blood Test (FOBT) or Faecal Immunochemical Test (iFIT)

*Repeat for **each** test indicated as yes:*

14) When is this test booked for?

- ☐ Within the next month
- ☐ In 1-2 months time
- ☐ In 3-4 months time
- ☐ In more than 4 months time

*Repeat for **each** test indicated as yes:*

15) What is the purpose of the test?

- ☐ Don’t know/not sure
- ☐ To see if surgery has removed all the bowel cancer
- ☐ To see if the cancer has spread
- ☐ To see if the cancer has come back
- ☐ To test whether I definitely had bowel cancer/diagnose bowel cancer

Demographics

For this final section I would like to ask you a few questions about you and your background.

1) Could you tell me your age?

i) *Record response*

2) What is the highest level of schooling you have completed? *Read out as necessary, only code one answer.*

- University degree
- TAFE of trade certificate or diploma
- Year 12 or Higher School Certificate
- Year 10 or School Certificate
- Primary School
- Other, please specify _________________
- Refused (*DO NOT READ OUT)*

3) What is your current marital status?

Read out as necessary

- Married
- Living in a defacto relationship
- Divorced
- Married, but separated
- Widowed
- Never married
- Refused (*DO NOT READ OUT)*

4) How would you best describe your employment situation at the moment?

Read out as necessary

☐ Employed full time

☐ Employed part time/casual

☐ Unemployed (not retired or on pension) go to 6

☐ Student (full time or part time) go to 6

☐ Retired go to 6

☐ Permanently unable to work/ill go to 6

☐ Other – Please describe go to 6

☐ Refused (*DO NOT READ OUT)* *go to 6*

5) What is your usual occupation?

i) *Record response*

6) Do you have private health cover? *If yes:* **Is that hospital, extras or package cover?**

☐ Yes

☐ No
