# Supplementary material for: Improving adherence to colorectal cancer surveillance guidelines: results of a randomised controlled trial
Source: BMC Cancer. 2017 Feb 6;17:106. doi: 10.1186/s12885-017-3095-x (PMC5294678; doi:10.1186/s12885-017-3095-x)
Supplement: Additional file 2: — Follow up survey sent to participants 12 months after the baseline interview. Description: Questions from the pen and paper follow up survey used in the research presented. (PDF 24 kb) [file 12885_2017_3095_MOESM2_ESM.pdf]

12 month Follow-up survey

Bowel cancer follow-up care

1. Over the past 12 months have you seen your cancer specialist for follow up care?

- ☐ Yes
- ☐ No — please go to question 4

2. How many times in the last 12 months have you visited your cancer specialist for follow up care?

________times

3. Over the past 12 months have you visited your GP for bowel cancer follow up care? This could include management of ongoing symptoms, discussion of symptoms of concern, or follow up tests.

- ☐ Yes
- ☐ No — please go to question 5

4. How many times in the last 12 months have you visited your GP for bowel cancer follow up care?

________times

5. Colonoscopy. This is usually a day procedure in hospital where the inside of your colon is examined while you are sedated.

Have you had a colonoscopy in the past 12 months?

- ☐ Yes
- ☐ No — please go to question 8

6. What month did you have the colonoscopy in?

☐ January	☐ July
☐ February	☐ August
☐ March	☐ September
☐ April	☐ October
☐ May	☐ November
☐ June	☐ December

7. Did you have any days off work or days when you could not do your usual activities due to having the colonoscopy?

- ☐ No
- ☐ Yes, 1 day
- ☐ Yes 2-3 days
- ☐ Yes, 4-7 days
- ☐ More than 7days.

8. Faecal occult blood test (FOBT) / faecal immunochemical test (FIT) or immunochemical faecal occult blood test (iFOBT). For any of these tests you would have been asked to provide samples of faeces or poo. The samples would have been tested for tiny amounts of blood. Certain foods and medications can affect some of these tests, so you may have been asked not to eat red meats or large amounts of vitamin C in the days before having the test.

Have you had an FOBT, FIT or iFOBT in the past 12 months?

- ☐ Yes
- ☐ No — please go to question 11

9. What month did you have the FOBT, FIT or iFOBT in?

☐ January	☐ July
☐ February	☐ August
☐ March	☐ September
☐ April	☐ October
☐ May	☐ November
☐ June	☐ December

10. Did you have any days off work or days when you could not do your usual activities due to having the FOBT, FIT or iFOBT?

- ☐ No
- ☐ Yes, 1 day
- ☐ Yes 2-3 days
- ☐ Yes, 4-7 days
- ☐ More than 7 days.

11. Sigmoidoscopy. In this procedure only the rectum and lower part of the colon are examined. This is a short procedure which lasts about 5-10 minutes. Sedation is not usually required and you can usually go straight home after the procedure.

Have you had a sigmoidoscopy in the past 12 months?

- ☐ Yes
- ☐ No please go to question 14

12. What month did you have the sigmoidoscopy in?

☐ January	☐ July
☐ February	☐ August
☐ March	☐ September
☐ April	☐ October
☐ May	☐ November
☐ June	☐ December

13. Did you have any days off work or days when you could not do your usual activities due to having the sigmoidoscopy?

- ☐ No
- ☐ Yes, 1 day
- ☐ Yes 2-3 days
- ☐ Yes, 4-7 days
- ☐ More than 7days.
